# Decoding respiratory syncytial virus morphology: distinct structural and molecular signatures of spherical and filamentous particles

**DOI:** 10.3389/fcimb.2025.1597279

**Published:** 2025-05-27

**Authors:** Manoj K. Pastey, Lewis H. McCurdy, Barney Graham

**Affiliations:** ^1^ Department of Veterinary Biomedical Sciences, Oregon State University, Corvallis, OR, United States; ^2^ Department of Infectious Diseases, Wake Forest University School of Medicine, Charlotte, NC, United States; ^3^ Vaccine Research Center, National Institute of Allergy and Infectious Diseases (NIAID), National Institutes of Health, Bethesda, MD, United States

**Keywords:** respiratory syncytial virus, spherical particles, filamentous particles, morphology, sucrose gradient velocity sedimentation

## Abstract

Respiratory syncytial virus (RSV) is a pleomorphic enveloped virus that buds as both spherical and filamentous particles. The determinants of RSV particle morphology and the roles of these forms in transmission and pathogenicity are not clearly defined, owing to a complex interplay of viral proteins and host factors that remains poorly understood. To further characterize RSV morphology, we developed a sucrose gradient velocity sedimentation method to separate spherical and filamentous virions. Fluorescence microscopy and electron microscopy (EM) confirmed two distinct peaks containing predominantly spherical or filamentous particles, respectively. Notably, EM images revealed a distinctive “honeycomb” pattern on the RSV envelope, suggesting an ordered lattice of glycoproteins on the virion surface. Biochemical analyses of viral protein and lipid content showed that filamentous particles contained higher levels of uncleaved fusion protein F0 and exhibited distinct phospholipid profiles compared to spherical particles. Both forms were enriched in cholesterol and phospholipids characteristic of lipid rafts, consistent with the idea that RSV buds from lipid raft microdomains. This enrichment in raft lipids is linked to cell-to-cell fusion (syncytium) formation and virion assembly. Quantitative real-time PCR analysis indicated a high particle-to-PFU ratio (~4:1), meaning only about one in four RSV virions was infectious. Spherical particles contained on average ~3 genomic RNA copies per virion, whereas filamentous particles contained ~2 copies. These data reveal several structural and compositional differences between RSV particle morphologies that may influence viral pathogenesis, and they provide a foundation for new antiviral approaches targeting virion assembly and morphology.

## Introduction

Respiratory syncytial virus (RSV) is a leading cause of acute lower respiratory tract infections, especially in infants, young children, and the elderly (Mufson et al., 1973; WHO, 2025). Globally, RSV is responsible for ~3.6 million hospitalizations and about 100,000 deaths in children under 5 each year (WHO, 2025). It also causes significant morbidity and mortality in older adults (WHO, 2025). RSV is an enveloped, negative-sense RNA virus (family *Pneumoviridae*) that exhibits pleomorphic virion morphology, budding from infected cells as both spherical and filamentous particles. Spherical RSV virions are typically 150–300 nm in diameter, whereas filamentous forms can be as thin as 60–100 nm and extend up to 10 μm in length (Berthiaume et al., 1974; Roberts et al., 1995). Filamentous morphologies are also observed in other respiratory viruses, such as influenza and parainfluenza viruses, and their emergence may be modulated by viral structural genes and host factors including the cytoskeleton and lipid microdomains (Roberts et al., 1998). In the case of influenza A virus, the matrix protein M1 is a principal determinant of virion shape (Shaikh et al., 2012). By contrast, RSV filament formation appears to rely primarily on viral proteins (e.g., the matrix protein M) and can occur even when actin polymerization is inhibited (Shaikh et al., 2012) highlighting that the exact molecular determinants of RSV morphology remain to be elucidated (Shaikh et al., 2012). Previous studies have shown that activation of the host small GTPase RhoA and the incorporation of RSV assembly sites into cholesterol-rich lipid rafts promote the formation of filamentous virus and the development of syncytia (Gower et al., 2001; McCurdy and Graham, 2003). RhoA signaling in infected cells facilitates the formation of long viral filaments that protrude from the cell surface and disrupting RhoA or depleting membrane cholesterol results in shorter or absent filaments and reduced cell–cell fusion (Gower et al., 2005). Selective budding from lipid raft microdomains appears to drive filamentous virion assembly in microvilli, which is associated with enhanced cell-to-cell spread (syncytium formation) (Gower et al., 2005). Thus, while RSV’s pleomorphic nature is well recognized, the precise viral and host factors governing whether RSV buds as a filament or a sphere are not well defined and require further investigation (Shaikh et al., 2012).

Understanding how virion morphology influences RSV infection and pathogenesis is important because different particle forms may play distinct roles during infection. Filamentous virions can remain attached to infected cells and form physical bridges to neighboring cells (Gower et al., 2005), enabling direct cell-to-cell transmission of infection without diffusing through extracellular space. This mode of spread via syncytium formation likely helps RSV evade certain immune defenses, such as neutralizing antibodies, by avoiding exposure of virions to the extracellular environment (Bitko et al., 2003; Partlow et al., 2025). Indeed, cell fusion (syncytium formation) is considered an efficient mechanism of viral dissemination between cells (Bitko et al., 2003). Spherical virions, in contrast, are more compact and may be better suited for cell-free transmission (for example, in respiratory droplets or aerosols) due to their smaller size and use of fewer resources per particle (Partlow et al., 2025). Knowledge of these morphology-specific roles is important for vaccine development and antiviral strategies, as interventions might need to target both forms. For instance, if filamentous particles preferentially mediate cell-to-cell spread and immune evasion, antiviral approaches that disrupt filament formation could limit RSV’s ability to form syncytia and persist in host tissues. Likewise, a better understanding of RSV morphology could inform manufacturing of live-attenuated vaccines by ensuring the production of the most immunogenic or transmissible form.

In this study, we set out to isolate and characterize the spherical and filamentous forms of RSV and to compare their structural and molecular features. We developed a velocity sedimentation approach to separate RSV particles by morphology and then analyzed each population’s protein composition, lipid composition, and genome content. By defining distinct signatures of spherical vs. filamentous RSV, we aim to shed light on how virion morphology might influence viral infectivity, pathogenicity, and interaction with the host. These insights could provide the basis for novel antiviral strategies that target virion assembly or exploit the vulnerabilities associated with each morphological form.

## Materials and methods

### Virus and cell culture

RSV strain A2 (subgroup A) was obtained from Dr. Robert Chanock (NIH, Bethesda, MD) and propagated in HEp-2 cells as described previously (Graham et al., 1988). HEp-2 cells (ATCC CCL-23) were maintained in Eagle’s Minimum Essential Medium (EMEM) supplemented with 10% fetal bovine serum (FBS), 2 mM L-glutamine, 50 μg/mL gentamicin, and 100 U/mL penicillin G, at 37°C in a 5% CO_2_ atmosphere.

### Virus infection and harvest

HEp-2 cell monolayers in T-162 flasks (growth area 162 cm²) were infected with RSV at a multiplicity of infection (MOI) of 0.5. The virus was allowed to adsorb for 1 hour at 37°C, then fresh culture medium was added. After 72 hours of incubation (when extensive cytopathic effect was observed but before complete cell detachment), the infected cell cultures were harvested for virus purification.

### Sucrose gradient separation of RSV particles

To separate spherical and filamentous RSV particles, we developed a velocity sedimentation method using a continuous sucrose gradient. Infected cell suspensions (including cells and supernatant) were layered onto a 15–60% (w/v) continuous sucrose gradient prepared in TM buffer (50 mM Tris, 10 mM MgSO_4_, pH 7.4). Gradients were centrifuged at 14,000 rpm for 10 minutes at 4°C in a Sorvall SureSpin 630 swinging-bucket rotor. Twelve 2-ml fractions were carefully collected from the top of the gradient. Each fraction was analyzed for infectious virus by plaque assay on HEp-2 cells (see below) to identify peaks of viral infectivity.

### Plaque assay

RSV titers in gradient fractions were determined by plaque assay on HEp-2 cell monolayers (80% confluence in 12-well plates), as described by Graham et al. (1988). Serial ten-fold dilutions of each sample were prepared in EMEM and applied to cells for 2 hours at 37°C. The inoculum was then removed, and cells were overlaid with 0.8% methylcellulose in maintenance medium. After 5 days of incubation, monolayers were fixed with 10% formalin and stained with 1% crystal violet to visualize plaques. Plaque-forming units (PFU) per ml were calculated for each fraction.

### Concentration of peak fractions

Fractions corresponding to the major infectivity peaks were pooled or processed individually as needed for downstream analysis. To concentrate virus particles from these fractions, samples were subjected to ultracentrifugation. Each selected fraction (~2 mL) was diluted in TM buffer and pelleted at 14,000 rpm (approximately 20,000 × *g*) for 10 minutes at 4°C in a Beckman ultracentrifuge. The virus pellet was resuspended in a small volume (50–100 µL) of TM buffer for further analyses.

### Immunofluorescence microscopy

Aliquots of the concentrated peak fractions were used for immunofluorescence microscopy to visualize virion morphology. A 50 µL drop of each sample was smeared onto a glass coverslip and allowed to air dry. The attached virus particles were fixed by incubation in 3.7% formaldehyde in phosphate-buffered saline (PBS) for 30 minutes at room temperature. After three rinses in PBS, samples were blocked with 5% non-fat dry milk in PBS for 30 minutes. RSV particles on the coverslip were stained with a mouse monoclonal antibody against the RSV fusion glycoprotein F (Maine Biotechnology Services) as the primary antibody (detects both cleaved and uncleaved F). After washing, samples were incubated with rhodamine-conjugated goat anti-mouse IgG (H+L) secondary antibody (ThermoFisher) for 1 hour. Following three washes with PBS containing 0.1% Tween-20, the coverslips were mounted on glass slides with Fluoromount-G. Specimens were examined using a Zeiss AxioPlan 2 fluorescence microscope. Images were captured with a Hamamatsu ORCA-ER digital camera and processed using Zeiss imaging software.

### Transmission electron microscopy

To confirm particle morphology, gradient peak fractions were examined by transmission electron microscopy (TEM) (Advanced Biotechnologies Inc., Columbia, MD). Sample aliquots (20 µL) from the spherical and filamentous peaks were deposited on carbon-coated 300-mesh gold grids. After 5 minutes, excess liquid was blotted, and the grids were immediately negatively stained with 2% phosphotungstic acid (pH ~7.0) for 30 seconds. Grids were air-dried and then observed with a JEOL 1200EX TEM at an accelerating voltage of 80 kV. For size calibration, 112 nm latex beads were included as a reference on some grids. Digital micrographs were taken at 50,000–65,000× magnification. Particle lengths and diameters were measured from TEM images to distinguish spherical vs. filamentous forms.

### Western blot analysis of viral proteins

The protein composition of RSV in each fraction was analyzed by SDS-PAGE and Western blot. Gradient fractions (1–12) were first concentrated by ultracentrifugation as described above. Each virus pellet was resuspended in lysis buffer (M-PER Mammalian Protein Extraction Reagent, Pierce Biotechnology) and the total protein content was quantified using a bicinchoninic acid (BCA) protein assay (Pierce). Equal amounts of viral protein from each fraction were mixed with Laemmli sample buffer, boiled, and separated on a 10% SDS-PAGE gel. Proteins were transferred to PVDF membranes (Millipore). The blots were probed with a rabbit anti-RSV polyclonal antibody (Chemicon) that detects multiple RSV structural proteins, followed by an HRP-conjugated goat anti-rabbit secondary antibody (GE Amersham). Bound antibodies were detected using an enhanced chemiluminescence (ECL) substrate (Amersham Pharmacia Biotech). Chemiluminescent signals were captured on X-ray film. For quantification, digital images of the developed films were analyzed using ImageJ software. Densitometry was performed on each lane to measure relative band intensities corresponding to specific RSV proteins (the viral fusion protein F0/F1 and major internal proteins N, P, M). Care was taken to ensure signals were in the linear range of detection; background was subtracted, and no additional brightness/contrast adjustments were applied prior to analysis.

### Phospholipid analysis by HPLC–mass spectrometry

Lipid extraction and analysis were performed to compare the envelope lipid composition of spherical vs. filamentous RSV. Viral membrane lipids were extracted from purified particle preparations (pooled peak fractions) using the Folch method (Folch et al., 1957), which partitions lipids into a chloroform-methanol phase. The extracted lipids were dried under nitrogen and re-dissolved in chloroform: methanol (2:1) containing a mixture of deuterated internal standards for quantification. Lipid samples were analyzed by reversed-phase high-performance liquid chromatography coupled to electrospray ionization mass spectrometry (HPLC-ESI-MS), as described by Kim et al. (1994). Briefly, samples were injected onto a C18 analytical column (150 × 2.0 mm, 5 µm particle, Phenomenex, Torrance, CA). Phospholipid molecular species were separated using a gradient mobile phase of water and methanol-hexane (with 0.5% ammonium hydroxide) and detected with a Hewlett-Packard 1100 MSD mass spectrometer. Key phospholipid classes analyzed included sphingomyelins (SM), phosphatidylserines (PS), phosphatidylcholines (PC), and phosphatidylinositols (PI). Peak integration and quantification were done against the internal standards to determine the relative abundance of specific lipid molecular species in each sample.

### RNA extraction and quantitative real-time PCR

To determine the viral genomic RNA content in each virus population, we performed quantitative real-time RT-PCR targeting the RSV N gene. Viral RNA was extracted from the purified peak fractions (spherical and filamentous) using RNAzol-B reagent (Isotex Diagnostics) according to the manufacturer’s protocol. Extracted RNA was resuspended in RNase-free water and quantified by spectrophotometry. For cDNA synthesis, 1 µg of viral RNA from each sample was reverse transcribed using Superscript II reverse transcriptase (Invitrogen) with an RSV N gene-specific primer (N2, see below). The resulting cDNA was treated with RNase H and used as template for PCR. Primers and probe were selected based on published sequences (Hu et al., 2003) to specifically detect RSV nucleocapsid (N) gene RNA. The forward primer N1 (positions 19–45 of the N gene, 5′-GCTCTTAGCAAAGTCAAGTTGAATGA-3′) and reverse primer N2 (positions 81–101, 5′-TGCTCCGTTGGATGGTGTATT-3′) amplify a 63-nt segment. The TaqMan probe NPB48 (positions 47–79, 5′-ACACTCAACAAAGATCAACTTCTGTCATCCAGC-3′) was labeled with 6-FAM at the 5′ end and TAMRA at the 3′ end. Primers were synthesized by Integrated DNA Technologies and the probe by Applied Biosystems. Quantitative PCR was performed in 50 µL reactions containing cDNA (equivalent to 0.05 µL of original sample RNA), Platinum Quantitative PCR SuperMix-UDG (Invitrogen), 0.5 µM of primers N1 and N2, and 0.2 µM probe. Amplification and real-time fluorescence detection were carried out on an MJ Research Opticon 2 thermocycler with the following cycling conditions: UDG incubation at 50°C for 2 min, initial denaturation at 95°C for 2 min, followed by 45 cycles of 95°C for 15 s and 60°C for 30 s. Fluorescence data were collected during the 60°C annealing/extension phase each cycle. Standard curves were generated using ten-fold serial dilutions of a plasmid containing the RSV N gene, to enable calculation of genome copy numbers in each sample. All reactions were run in triplicate, and no-template and no-RT controls were included to confirm specificity.

## Results

### Separation of spherical and filamentous RSV particles

**Figure 1:**
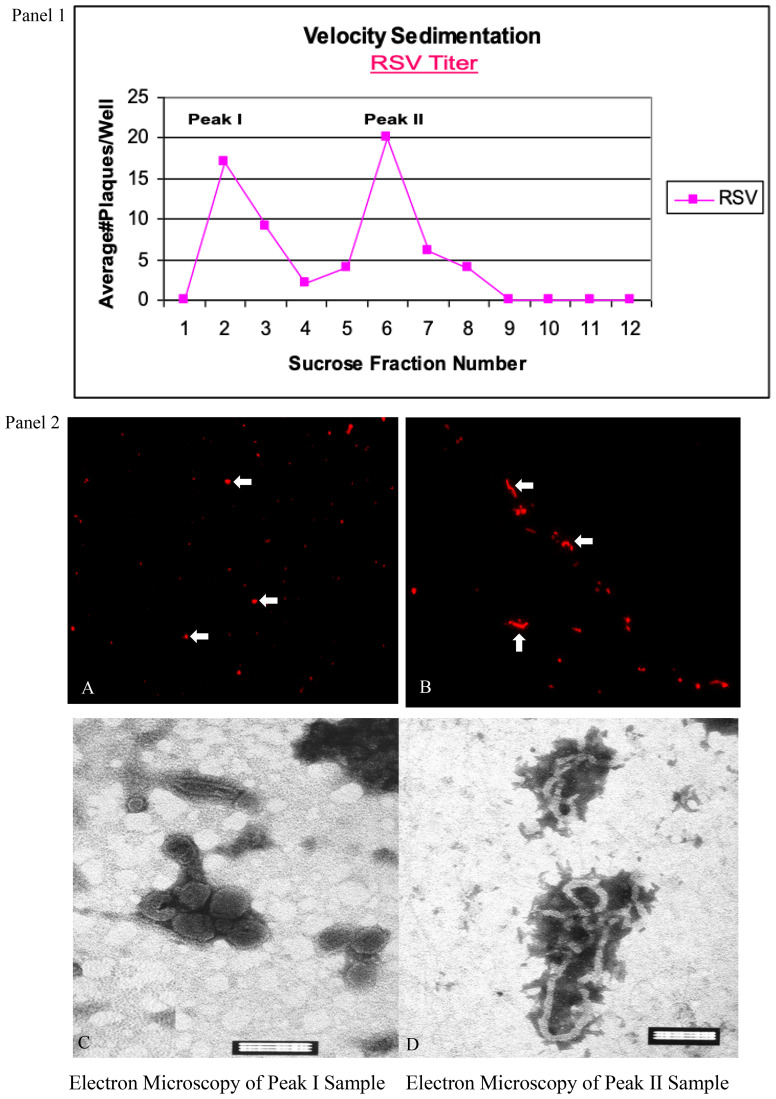
Separation of spherical and filamentous RSV particles by sucrose gradient velocity sedimentation. *Panel 1*: Infectivity profile of gradient fractions. RSV was grown for 72 h and loaded onto a 15–60% continuous sucrose gradient. Twelve fractions were collected after centrifugation. Plaque assays of each fraction (PFU/mL on the y-axis) revealed two distinct peaks of infectious virus (major peaks at fraction 2 and fraction 6). *Panel 2;* **(A, B)**: Immunofluorescence images of virus in peak fractions. Fraction 2 **(A)** shows mostly spherical particles (examples indicated by arrowheads), whereas fraction 6 **(B)** is enriched in filamentous particles (arrows). RSV particles were stained with anti-F monoclonal antibody and rhodamine-labeled secondary antibody (red). Scale bar = 10 µm. *Panel 2;* **(C, D)**: Transmission EM of peak fractions confirms particle morphology. **(C)** Representative spherical RSV virions from fraction 2 (diameters ~200 nm). **(D)** Representative filamentous RSV from fraction 6 (lengths ≥1 µm). Data represent three independent experiments yielding similar results. Scale Bar =100 nm **(C, D)**.

RSV grown in cell culture was successfully separated into two distinct populations by velocity sedimentation on a sucrose gradient. Traditional purification methods such as ultracentrifugation through discontinuous gradients (e.g., iodixanol) often yield mixed viral populations (Gias et al., 2008). In our continuous sucrose gradient (15–60%), two peaks of infectivity were observed in the plaque assay of gradient fractions, corresponding to fraction 2 and fraction 6 (**Figure 1**, Panel 1). Fraction 2 (upper band) and fraction 6 (lower band) contained the majority of viral PFUs, suggesting that RSV particles partition into two subsets by sedimentation rate. Fluorescence microscopy of these fractions stained for the F glycoprotein revealed that fraction 2 consisted predominantly of small, punctate particles consistent with spherical or short-filament virions, whereas fraction 6 was enriched in long filamentous particles (**Figure 1**, Panel 2; A, B). Transmission EM further confirmed the morphology: fraction 2 contained mostly spherical virions ~200 nm in diameter (**Figure 1**, Panel 2; C), while fraction 6 contained abundant filamentous virions ranging from 1–10 µm in length (**Figure 1**, Panel 2; D). Interestingly, at high magnification the viral envelope in both preparations displayed a regular patterned structure. In particular, the filamentous virions showed a unique “honeycomb” surface lattice, likely reflecting a symmetrical arrangement of the viral envelope glycoproteins (F and G) on the virion surface. This ordered “grid-like” array of surface spikes has not been previously described for RSV (Unpublished data). Further structural analysis (e.g., by cryo-electron tomography) will be needed to confirm this novel observation.

### Protein composition of RSV spherical vs. filamentous particles

**Figure 2:**
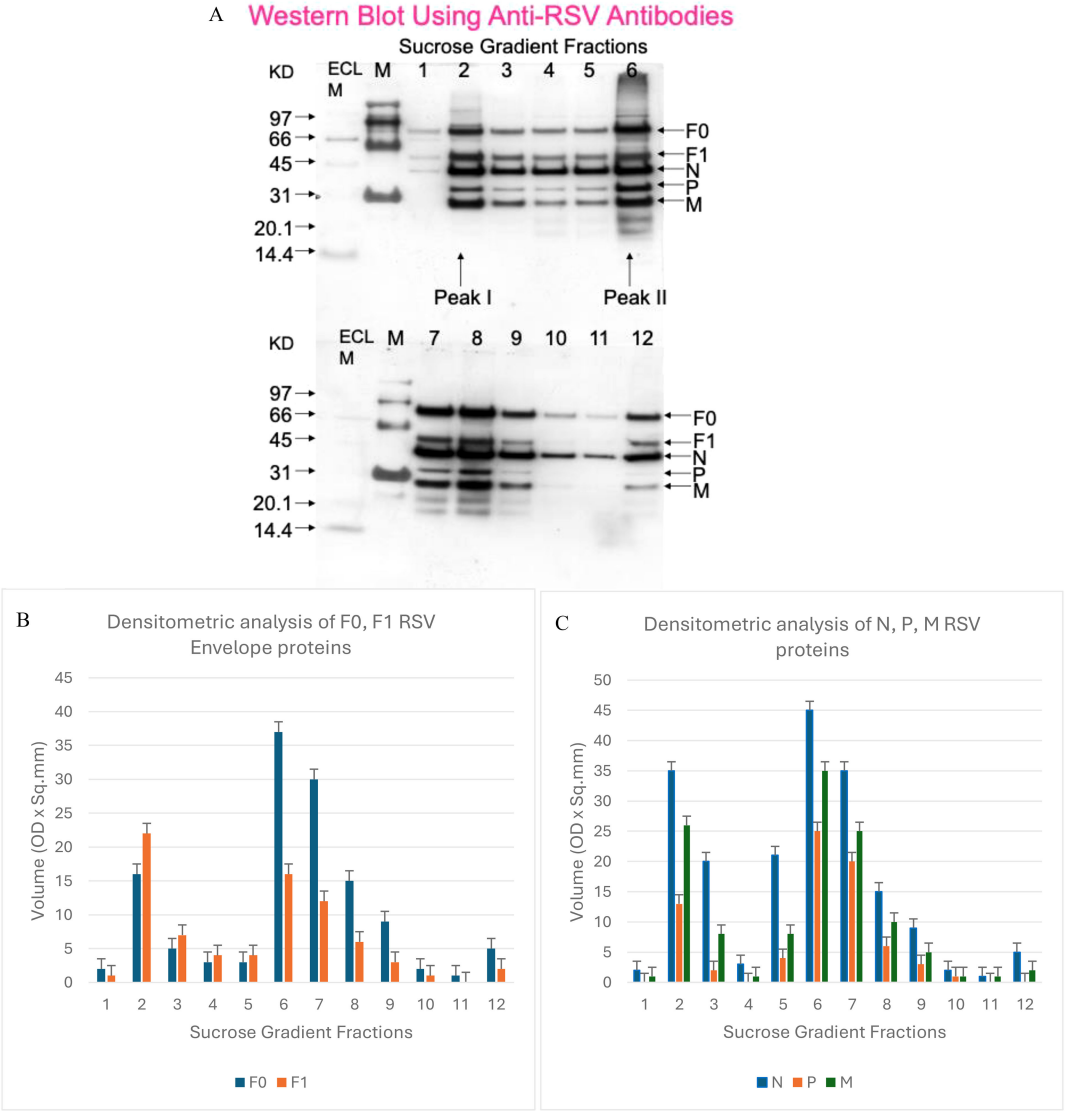
RSV protein composition in spherical versus filamentous virions. **(A)** Western blot of RSV proteins in gradient fractions 1–12. Blot was probed with anti-RSV polyclonal antibodies that detect major structural proteins. Key bands corresponding to F0 (unprocessed fusion protein), F1 (cleaved fusion protein subunit), N (nucleocapsid), P (phosphoprotein), and M (matrix) are indicated. **(B)** Densitometric analysis of envelope glycoproteins F0 and F1 in spherical (fraction 2) vs. filamentous (fractions 6–7) particles. The filamentous-rich fractions contain a higher proportion of uncleaved F0 relative to cleaved F1, whereas spherical particles have more F1. **(C)** Densitometry of internal proteins N, P, and M shows higher levels in filamentous particle fractions than in spherical. Data represent mean band intensity (arbitrary units) from three independent experiments (error bars indicate range).

We next compared the viral protein composition of the separated spherical and filamentous RSV populations. Limited information has been available in the literature regarding how the protein content of RSV might differ by virion morphology. To address this, we performed Western blot analysis on the gradient fractions using polyclonal antibodies that recognize RSV structural proteins. Viral proteins were detected across multiple fractions, but distinct patterns emerged for the two peaks. **Figure 2A** shows a representative blot of RSV proteins in fractions 1–12. Fractions 2 and 6 (the infectivity peaks) both contained the major RSV structural proteins, including the fusion protein F (present as uncleaved F0 and cleaved F1 subunits), the nucleocapsid protein N, the phosphoprotein P, and the matrix protein M, among others. Densitometric quantification of band intensities from three independent experiments is summarized in **Figures 2B, C**. We found that the uncleaved fusion protein precursor F0 was significantly more abundant (relative to total protein) in the filamentous particle fractions (peaking at fraction 6–7) than in the spherical particle fraction (fraction 2). Conversely, the cleaved F1 subunit of the fusion protein was relatively enriched in spherical particles. This indicates that filamentous RSV virions tend to carry more uncleaved F, whereas spherical virions carry more processed fusion protein, suggesting a difference in maturation or activation state of the fusion machinery between the two forms.

In addition, the major internal proteins showed quantitative differences: the N, P, and M proteins were all found at higher levels in fraction 6 (filamentous) compared to fraction 2 (spherical) (**Figure 2C**). For example, the matrix protein M, which forms the internal virion layer underlying the envelope, was present at notably higher amounts in filamentous virions. The greater abundance of these proteins in filamentous particles could simply reflect the larger size (and thus greater total protein content) of filaments. Indeed, a single filamentous virion (which can be several microns long) has a much greater mass than a ~200 nm spherical particle, so it would inherently contain more copies of each structural protein. Thus, the higher N, P, and M signals do not necessarily indicate a higher concentration of those proteins *per unit volume* of virion; rather, they likely scale with particle size. Interestingly, the phosphoprotein P exhibited a bimodal distribution, with peaks in both fraction 2 and fraction 6 that correlated with the highest infectious titers. This observation raises the possibility that P protein levels might be linked to infectivity, perhaps by influencing the assembly of the viral polymerase complex in new virions, although further studies would be needed to confirm any functional role.

Overall, our analysis confirmed that both spherical and filamentous RSV particles contain the full complement of viral structural proteins, but the relative representation of certain proteins (such as F0 vs F1, and the quantities of N/P/M) differs between the two morphologies. These protein composition differences could have functional implications. For instance, a higher content of uncleaved F0 in filaments might affect their fusion activity or stability, since F0 must be cleaved to F1 to trigger membrane fusion. The presence of more M protein in filaments might relate to the role of the matrix in assembling and maintaining a long filamentous structure; in other viruses like influenza, matrix oligomerization is critical for filament formation (Shaikh et al., 2012). Likewise, N and P are components of the nucleocapsid; their higher levels in filaments could reflect multiple genome copies packaged (see below) or a structural requirement for stabilizing the extended ribonucleoprotein in a filamentous virion.

### Lipid composition of RSV spherical vs. filamentous particles

Enveloped viruses like RSV acquire their membrane from the host cell during budding, and the lipid composition of the virion envelope can influence properties such as particle budding efficiency, stability, and cell entry. Many enveloped viruses preferentially bud from cholesterol-rich lipid rafts in the plasma membrane, which can concentrate viral glycoproteins and facilitate assembly (Ripa et al., 2021). RSV in particular has been reported to utilize lipid microdomains for assembly: filamentous RSV particles bud almost exclusively from lipid raft regions, whereas spherical particles can bud from both raft and non-raft regions (McCurdy and Graham, 2003). To examine whether the two forms of RSV have different lipid makeups, we performed quantitative lipidomics on purified spherical and filamentous virions (corresponding to peak fractions 2 and 6). Both forms of RSV showed lipid profiles characteristic of raft-derived membranes. In both spherical and filamentous particles, the relative content of cholesterol was high, and we observed enrichment of sphingomyelin (SM) and phosphatidylserine (PS), along with a corresponding lower proportion of phosphatidylcholine (PC) and phosphatidylinositol (PI), compared to typical non-raft cell membrane composition. These features confirm that both morphologies incorporate significant amounts of raft-associated lipids into their envelope, consistent with the idea that RSV assembly occurs in cholesterol-rich microdomains.

**Figure 3:**
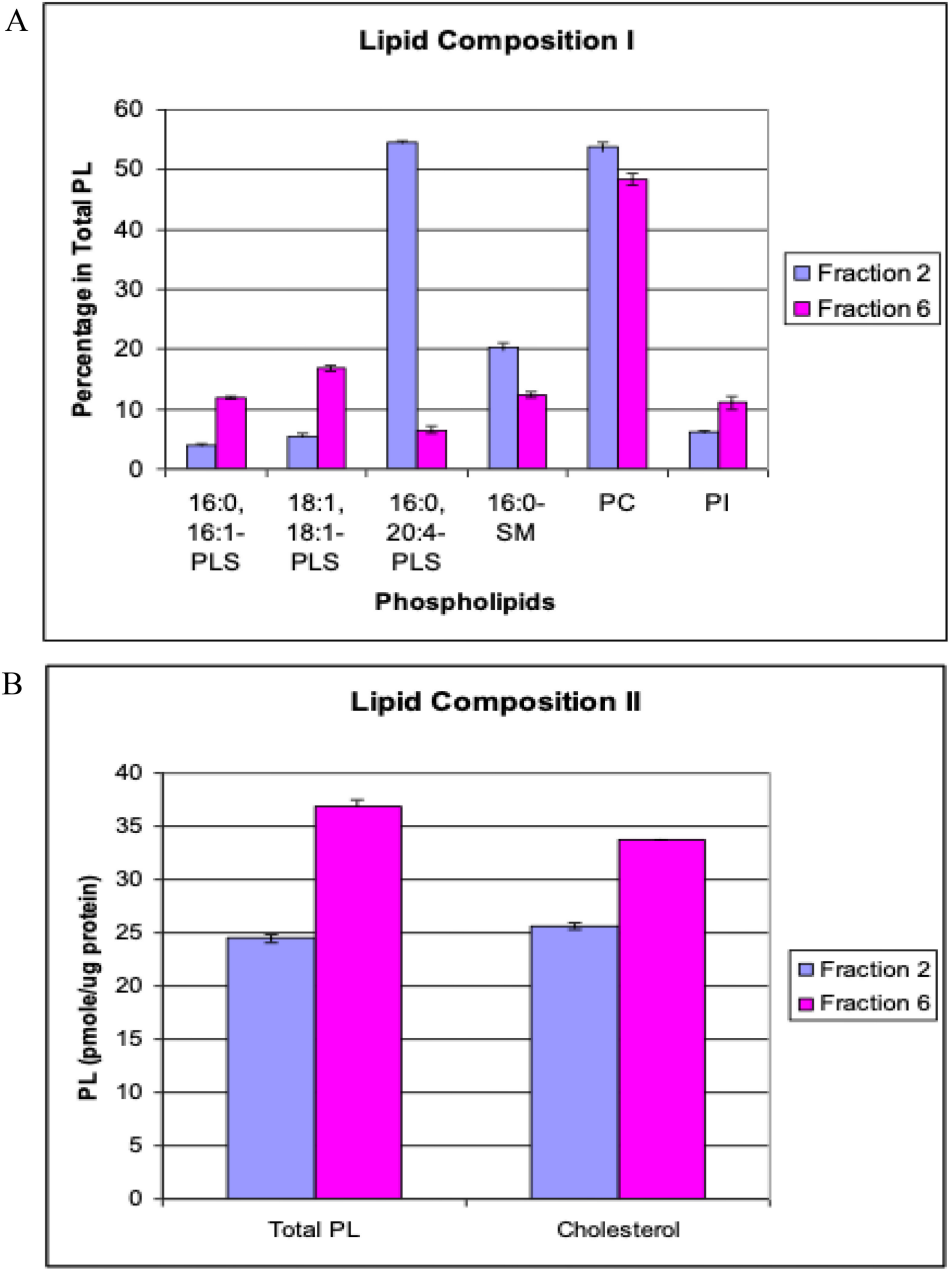
RSV lipid composition of spherical vs. filamentous virions. Lipids were extracted from RSV particles using the Folch method (Folch et al., 1957) and analyzed by reversed-phase HPLC-electrospray ionization mass spectrometry with a C18 column (Kim et al., 1994). Lipid extracts, spiked with d35-labeled phospholipids as internal standards, were directly injected onto the column and separated using a mobile phase containing water and 0.5% ammonium hydroxide in methanol-hexane. Detection was performed on a Hewlett-Packard HPLC-MS Series 1100 MSD instrument. **(A)** Specific phospholipid molecular species differentially enriched in spherical (blue bars) versus filamentous (red bars) RSV particles. Examples include distinct species of phosphatidylserine (PLS), sphingomyelin (SM), phosphatidylcholine (PC), and phosphatidylinositol (PI) that show statistically significant differences between the two forms (p < 0.05). **(B)** Comparison of total phospholipid and cholesterol content per particle (arbitrary units normalized to spherical virion = 1). Filamentous virions have a higher total lipid content, consistent with their larger membrane area. Data represent the average of three independent experiments. Abbreviations: SM, sphingomyelin; PS, phosphatidylserine; PC, phosphatidylcholine; PI, phosphatidylinositol.

Despite these overall similarities, we detected notable differences in specific phospholipid molecular species between spherical and filamentous RSV. **Figure 3A** summarizes the comparative lipidomic analysis. Spherical particles had higher levels of certain phospholipid species, for example: 16:0–20:4 PLS (a phosphatidylserine containing palmitic acid and arachidonic acid), 16:0 SM (palmitoyl sphingomyelin), and two particular PC species (18:1–20:2 PC and a mixed 18:1–22:4/18:0–22:5 PC). In contrast, filamentous particles showed elevated levels of other lipid species, including 16:0–16:1 PLS and 16:1–16:1 PLS (phosphatidylserines containing palmitic and palmitoleic or dipalmitoleic acids), 24:0 SM (lignoceroyl sphingomyelin), and a specific PI species (16:0–18:2/16:1–18:1 PI). These differences suggest that the two forms of RSV incorporate lipids in different proportions, which could affect the biophysical properties of the virion envelope. For instance, sphingomyelin and saturated fatty acids confer more ordered, less fluid membrane domains, which might help maintain the structural integrity of long filaments. Phosphatidylserine exposure on the outer leaflet of viral envelopes is known to influence immune recognition, as some viruses exploit PS to bind host receptors (e.g., TIM/TAM family) and dampen immune responses (Meertens et al., 2012; Wang et al., 2021). If filamentous RSV particles have a different PS content or distribution, this could potentially modulate how they interact with phagocytic cells or complement compared to spherical particles.

When comparing overall lipid quantity, filamentous RSV particles contained a greater total amount of phospholipid and cholesterol per virion than spherical particles (**Figure 3B**). Again, this is expected given the much larger membrane area of a filamentous virion. Importantly, however, the enrichment of certain lipid types in one morphology over the other might reflect adaptations to each form’s mode of assembly or function. For example, the unique set of lipid species found in filamentous virions might contribute to the curvature and flexibility needed for extremely elongated particles or might influence how these filaments fuse with cell membranes. Conversely, spherical virions, being smaller, might prioritize lipid species that maximize fusion efficiency or stability in extracellular environments.

### Genome packaging and infectivity of RSV particles

To evaluate how virion morphology might relate to genome packaging and infectivity, we quantified the number of RSV genome copies in spherical and filamentous particles and compared this to infectious titers.

**Figure 4:**
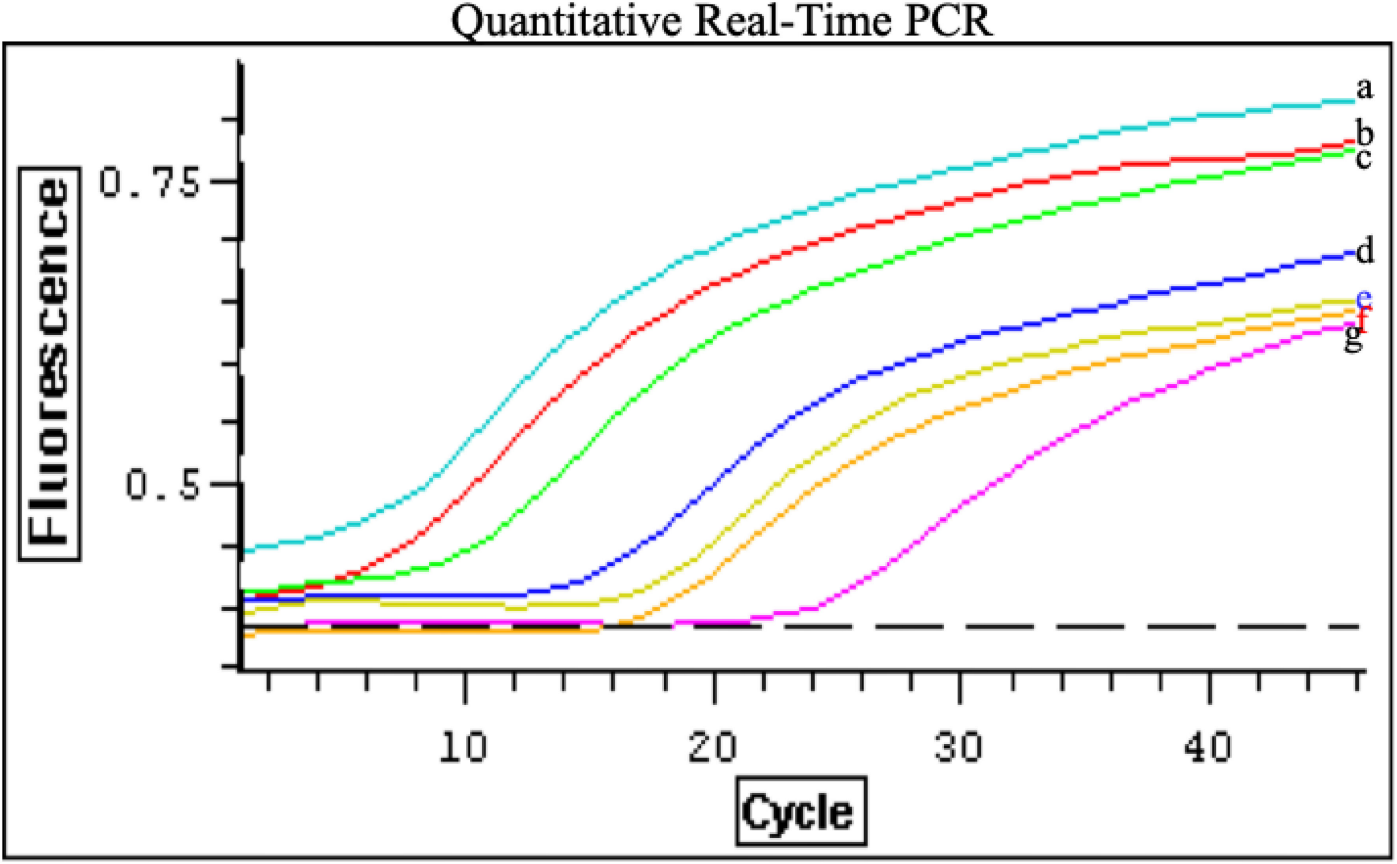
Genome copy number of RSV particles determined by quantitative real-time PCR: PCR reactions were carried out with 0.05 µl cDNA in a 50-µl reaction mixture containing Platinum Quantitative PCR SuperMix-UDG (Invitrogen), 0.5 µM N1 and N2 primers, and 0.2 µM probe, as recommended by the manufacturer. Real-time RT-PCR was performed using an Opticon 2 detection system (MJ Research). The cycling conditions were 50°C for 2 min (UDG incubation), 95°C for 2 min (UDG inactivation), followed by 45 cycles of 15 s at 95°C and 1 min at 60°C to amplify the RT products. Standard curve of quantitative real-time PCR for RSV N gene (positive control) was used to calculate genome copies in each sample. Negative controls and serial dilutions of positive controls (plasmid containing the N gene) were included in each PCR assay. Tenfold serial dilutions of the pTM-N plasmid were prepared, containing 4.8×10¹^10^ **(a)**, 4.8×10^9^ **(b)**, 4.8×10^8^ **(c)**, 4.8×10^6^ **(d)**, and 4.8×10³ **(g)** copies per reaction mixture. Additionally, 0.05 µl of reverse-transcribed cDNA from peak fraction 2 **(f)** and fraction 6 **(e)** was used per reaction, with RSV copy numbers of 3.8×10^5^ and 4.7×10^5^, respectively. Data represent three independent experiments.

- Genome copy number per particle: To quantitate the genome copy per particle, we compared particle count enumerated by electron microscopy from fraction 2 and fraction 6 to RNA copy numbers obtained by Taqman real-time PCR method targeting the N gene. The particle counts of spherical and filamentous were 1.26x10^5^ and 2.6x10^5^, respectively. The RNA copy number from quantitative real-time PCR were 3.8x10^5^ for spherical particles and 4.7x10^5^ for filamentous particles (**Figure 4**). Therefore, spherical RSV particles were found to contain on average approximately 3 genomic RNA copies per virion, whereas filamentous RSV particles contained about 2 copies per virion. This difference (3 vs 2 copies) was small but consistently observed across experiments. The presence of more than one genome per virion in both cases suggests that RSV, like many other RNA viruses, can package multiple ribonucleocapsids in a single envelope, especially in larger particles (Roberts et al., 1995, 1998). However, not all packaged genomes may be complete or functional. In fact, it is known that many enveloped RNA viruses produce a proportion of defective genomes during replication, leading to the formation of defective interfering (DI) particles that contain truncated or inoperative genomes (Lazzarini et al., 1981; Treuhaft and Beem, 1982). These DI particles cannot establish infection on their own but can interfere with standard virus replication when present.
- Genome copy number per plaque: RSV stability is influenced by temperature and buffer composition, potentially affecting infectivity. Previous studies suggest that filamentous RSV particles are more infectious than spherical ones (Roberts et al., 1995). To determine the number of viable virus and to compare the infectivity of spherical and filamentous particles, we quantitated the genome copy per plaque by comparing the number of plaques (infectious titer) from fraction 2 and fraction 6 to RNA copy numbers obtained by Taqman real-time PCR method. The plaque assay from fraction 2 and fraction 6 yielded 3.6x 10^7^ pfu/ml and 4.4 x 10^7^ pfu/ml, respectively. The RNA copy number obtained from fraction 2 and fraction 6 were 1.5 x 10^8^ and 1.9 x 10^8^ pfu/ml, respectively (data not shown). This indicates that approximately 1 in 4 particles (25% infectivity) of both morphological forms were infectious, with no significant difference in infectivity between spherical and filamentous particles. The implication is that both forms contribute to the total infectious pool of virus and that both carry a comparable proportion of non-infectious particles. These non-infectious particles likely include the DI virions or otherwise damaged virions that carry genomic RNA but cannot initiate infection (Lazzarini et al., 1981; Treuhaft and Beem, 1982). The presence of DI particles might play a role in RSV pathogenesis by modulating viral replication dynamics and evading immune responses, as has been proposed for other paramyxoviruses (Roberts et al., 1998).

## Discussion

Our study provides new insights into the distinct structural and molecular characteristics of RSV’s two virion forms – spherical and filamentous. By physically separating these morphologies and analyzing their content, we have clarified some differences that may underlie their roles in infection and transmission. The determinants of RSV morphology have been an open question, and our results begin to address why the virus might produce both forms.

One key finding is that filamentous RSV particles are enriched in certain viral components (like uncleaved F protein and matrix protein) and specific host-derived lipids compared to spherical particles. These differences likely reflect adaptations of the filamentous form to its mode of assembly and function. The RSV matrix (M) protein in particular appears to be more abundant in filamentous virions, which aligns with the idea that robust M self-assembly is required to support the elongated filament structure (Shaikh et al., 2012). In related viruses such as influenza, mutations that disrupt matrix oligomerization prevent filament formation (Shaikh et al., 2012). It is plausible that RSV filaments similarly depend on a helical or lattice-like arrangement of M protein along their length, as suggested by recent cryo-ET studies (Ke et al., 2018) that visualized an organized matrix layer inside RSV filaments. The higher content of F0 (uncleaved fusion precursor) in filaments is also intriguing. Uncleaved F0 is fusion-inactive until proteolytically cleaved to F1 and F2, but its presence might stabilize the virion or modulate fusion timing. Perhaps filamentous particles, which often remain attached to the cell, retain more F0 to avoid premature triggering of fusion, whereas spherical particles (destined to be released and infect new cells) carry more readily activatable F1. This is speculative, but differential F protein cleavage could influence how each particle type mediates membrane fusion with target cells.

The enrichment of cholesterol and raft-associated lipids in both particle types reinforce the model that RSV buds from lipid raft microdomains of the plasma membrane (McCurdy et al., 2003). These microdomains not only concentrate the viral glycoproteins F and G but also host proteins like RhoA and CD44 that have been implicated in filament formation (McCurdy et al., 2003). The linkage between lipid rafts and syncytium formation is particularly noteworthy: RhoA-mediated actin reorganization leads to the formation of microvilli rich in raft lipids, and RSV filaments bud from these structures, effectively forming viral bridges between cells (Gower et al., 2005). By assembling in lipid rafts, RSV may ensure that the nascent virions (especially filaments) are in optimal locations to initiate fusion with adjacent cells. Our observation that filamentous virions have a somewhat unique lipid signature (e.g., more saturated phospholipids and specific sphingomyelins) suggests their membranes may be more ordered. A more ordered, rigid envelope could be advantageous for filaments, lending them the structural stability to protrude from cells without breaking. It could also influence their interaction with the host’s immune system. For instance, higher levels of phosphatidylserine in virion envelopes might enable RSV to engage PS-recognizing receptors on phagocytic cells (a strategy some viruses use to mimic apoptotic cell debris and dampen immune activation) (Meertens et al., 2012; Wang et al., 2021). Whether RSV exploits such a mechanism remains to be determined, but differences in lipid composition could conceivably affect how efficiently the immune system detects or neutralizes spherical vs. filamentous virions.

Our study also touches on the long-standing observation that many RSV particles are non-infectious. We quantified a particle-to-PFU ratio of ~4:1 for both forms, meaning roughly 75% of particles did not result in productive infection. This high ratio is not unusual for RSV and other paramyxoviruses, which commonly produce defective interfering genomes during replication (Lazzarini et al., 1981; Treuhaft and Beem, 1982). These DI particles, while not infectious by themselves, can modulate infection dynamics by competing for resources and by stimulating innate immune responses. The fact that both spherical and filamentous RSV had similar infectivity ratios suggests that both forms likely include a mix of standard and defective particles. It is interesting to consider whether the morphology could influence the generation or packaging of defective genomes. One could speculate that the larger volume of filaments might allow them to accommodate more genomic RNA segments, potentially increasing the chance of including a defective RNA along with a full genome. However, our data showed filaments actually had slightly fewer genomes on average than spheres (2 vs 3), which might indicate a more selective packaging or a requirement for maintaining a certain genome density. Regardless, the end result is that each form delivers about one viable genome per several particles. This parity in infectivity implies that the predominance of one form or the other in a given context (e.g., *in vivo*) could influence viral load and spread without one form being inherently more infectious than the other on a per-particle basis.

### Implications for pathogenesis

The ability of RSV to form filamentous virions appears to confer advantages for within-host spread. Filamentous RSV can form networks of infected cells via membranous bridges (large syncytia) (Gower et al., 2005), which may help the virus disseminate across the respiratory epithelium while evading neutralization by antibodies present in mucus or extracellular fluid. This mode of direct cell-to-cell transmission is reminiscent of other viruses that induce syncytia (e.g., measles virus, human metapneumovirus), and it can lead to extensive infection foci in tissues. Indeed, our understanding of RSV disease suggests that syncytium formation contributes to tissue pathology and persistence of the virus in the lungs. On the other hand, spherical particles, which are efficiently released into extracellular spaces, are likely important for initiating new infections in distal sites within the host or transmitting to new hosts via respiratory droplets. Spherical virions, being smaller, might penetrate mucus barriers more easily during aerosol transmission. Studies on influenza have shown that pleomorphic viruses can adopt different shapes under different conditions: when conditions favor cell-free transmission, smaller particles dominate, whereas under immune pressure or other stresses, filamentous forms increase (Partlow et al., 2025). Although RSV is different from influenza, it is tempting to speculate that RSV might similarly modulate its shape distribution in response to the host environment. For example, early in infection when spreading within the host, filamentous forms may be favored to maximize local cell-to-cell spread and immune evasion (Partlow et al., 2025). Later, or in the upper airway where virus exit is imminent, more spherical particles might be produced for efficient aerosolization. This “mixed-strategy” of pleomorphism could be a way for RSV to balance between maximizing replication within a host and transmission to the next host, as has been suggested for other pleomorphic viruses (Partlow et al., 2025).

### Immune evasion considerations

The structural differences we observed could impact how the immune system recognizes RSV. The honeycomb-like arrangement of glycoproteins on filamentous RSV, for instance, might affect the exposure of neutralizing epitopes on the F and G proteins. If the glycoproteins are packed more closely or in an ordered array, certain epitopes might be less accessible to antibodies. Some recent work on RSV has indicated that changes in virion shape (for example, when the matrix dissociates and filaments collapse into spheres) can alter the efficacy of complement deposition by antibodies (Kuppan et al., 2021). In line with this, one could imagine that the filamentous form, with its intact matrix and ordered surface, might present a more challenging target for the immune system, whereas the more “loose” arrangement on spherical particles could be easier for antibodies to bind. Moreover, syncytium formation itself is a form of immune evasion: by spreading directly from cell to cell, RSV reduces the time it spends exposed outside cells. Our data reinforce the connection between lipid rafts, filaments, and syncytia – all features that help RSV minimize exposure to neutralization. It has been observed that filamentous virions of some viruses are more resistant to neutralizing antibodies or physical stresses (Partlow et al., 2025). Filamentous RSV could similarly be more resilient in certain contexts, which might help it persist in the face of host immune responses.

### Therapeutic and vaccine implications

The distinctions between RSV particle types open up potential strategies for intervention. One approach is to target the host factors that favor filament formation. Our findings and prior studies point to the RhoA signaling pathway as a critical driver of filamentous virion morphogenesis and cell fusion (Gower et al., 2001, 2005). Inhibiting RhoA or its downstream effectors (e.g., with small molecule inhibitors or peptides) can prevent the formation of filaments and syncytia (Gower et al., 2005). In fact, a RhoA-derived peptide has been shown to block RSV-induced syncytium formation in cell culture and significantly reduce viral titers in mice (Pastey et al., 2000), highlighting this pathway as a viable antiviral target. Another strategy is to disrupt the lipid raft platform that RSV requires for efficient budding. Treatments that deplete membrane cholesterol or otherwise disrupt lipid rafts (for example, cyclodextrins or statin drugs) have been found to inhibit RSV assembly and reduce the release of infectious virus (Gower et al., 2005). Our demonstration that both spherical and filamentous virions are raft-derived suggests that cholesterol-targeting interventions could broadly impair RSV production. Notably, one study reported that the cholesterol-lowering drug lovastatin can inhibit RSV filament formation and reduce viral spread *in vitro* (as indicated by a reduction in cell-associated virus transmission) (Ravi et al., 2013). Such findings align with our observations and propose a potential repurposing of statins or other raft-disrupting agents as adjunct therapies for RSV infection.

From a vaccine perspective, understanding virion morphology is relevant for antigen presentation. If filamentous and spherical particles present viral antigens differently, the immune response elicited by each might vary. Most laboratory preparations of RSV (for vaccines or challenge stocks) are produced in Vero or HEp-2 cell culture, where the proportion of filaments can vary with cell type and conditions. It may be important to ensure that vaccine candidates (live-attenuated or inactivated) induce immunity against antigens in both contexts—for instance, antibodies that can neutralize virus in the context of a cell-associated filament, not just free virions. Our identification of the honeycomb glycoprotein lattice might be a structural feature to exploit: if confirmed, regions of F or G involved in that lattice could be targets for broadly neutralizing antibodies or structure-based vaccine design.

In conclusion, this work advances our understanding of RSV’s pleomorphic nature by delineating how spherical and filamentous virions differ in composition and potential function. The structural heterogeneity of RSV is not merely a curiosity; it has tangible effects on viral transmission, immune evasion, and pathogenicity. Filamentous virions, enriched in matrix and raft lipids, appear tailored for direct cell-to-cell spread and persistence in tissues, whereas spherical virions excel in cell-free dissemination and initiating new infection foci. Future research should build on these findings to uncover the precise molecular mechanisms that govern RSV morphology—such as the roles of specific RSV proteins (F, G, M) and host factors (RhoA, cytoskeletal elements, membrane domains) in deciding filament vs. sphere. It will also be valuable to investigate RSV morphology *in vivo* in respiratory tract tissues, to see how these forms contribute to disease severity or tropism. Ultimately, a deeper understanding of RSV particle morphology could inform novel antiviral interventions, such as drugs that tip the balance towards a less pathogenic form, or vaccines that effectively target the virus in all its forms. By “decoding” RSV morphology, we open the door to potentially disrupting a key aspect of the RSV life cycle that has so far been an elusive target.

## Data Availability

The raw data supporting the conclusions of this article will be made available by the authors, without undue reservation.
